# Effects of Physical Exercise on Working Memory and Attention-Related Neural Oscillations

**DOI:** 10.3389/fnins.2020.00239

**Published:** 2020-03-31

**Authors:** Alondra Chaire, Andreas Becke, Emrah Düzel

**Affiliations:** ^1^Institute of Cognitive Neurology and Dementia Research, Otto von Guericke University Magdeburg, Magdeburg, Germany; ^2^German Centre for Neurodegenerative Diseases, Magdeburg, Germany

**Keywords:** physical exercise, alpha power, visual attention, EEG, young adults

## Abstract

Cognitive functions, such as working memory (WM) and attention, have been shown to benefit from physical exercise. Quantifying frequency-band-specific neural oscillatory patterns during the use of such cognitive functions can provide insight into exercise-induced benefits in the brain. Specifically, we investigated whether a 4-month physical exercise training influenced theta and alpha power measured in visual WM and attention tasks. The delayed match-to-sample (DMS) task required mnemonic discrimination of similar visual stimuli, akin to pattern separation, while the visual-attention search (VAS) task required detecting the presence of a specific object (i.e., target) in an image. Behavioral and electroencephalographic data were acquired during a DMS visual WM task and VAS task both before and after the intervention. Forty-three sedentary young adults (19–34 years) were pseudorandomly assigned to a training group (indoor treadmill, *n* = 20) or to a control group (*n* = 23). Compared to the preintervention baseline, the exercise group showed increased frontal alpha power (9–12 Hz) during the VAS task after the intervention. In addition, alpha power changes correlated positively with fitness changes. Behaviorally, there were no exercise-related effects on reaction times or accuracy in either task. Our findings substantiate that aerobic training of sedentary young adults may influence neural dynamics underlying visual attention rather than visual WM and mnemonic discrimination.

## Introduction

The beneficial effects of exercise on brain function are of widespread interest but remain elusive. Human research has demonstrated that physical exercise training has the greatest impact on spatial memory, working memory (WM), and executive attention (Cassilhas et al., 2016; De Sousa et al., 2018). More specifically, other studies showed higher fit participants performed better during visual figure recognition (Maass et al., 2015), spatial memory (Erickson et al., 2009, 2011), and attentional control (i.e., the flanker task; Verstynen et al., 2012). However, exercise studies have been quite inconsistent, and the number of studies on chronic (i.e., long-term) physical exercise, especially in young adults, is limited (Verburgh et al., 2014). Hence, it is essential to continue to study the cognitive benefits and neural plasticity associations with exercise, particularly in young adults. Notably, very few studies obtained measures of neural function in a controlled exercise intervention in addition to the commonly obtained behavioral and structural measures.

It has been suggested that neural oscillations in the high- and low-frequency range, which can be measured by electroencephalogram (EEG), relate to distinct cortical operations. For instance, theta-band activity has been implicated in WM processes (Yamagishi et al., 2008) and has been shown to increase at frontal sites with task difficulty (Gundel and Wilson, 1992; Gevins et al., 1997), with WM load (Jensen et al., 2002) and with successful memory performance (Klimesch et al., 1996; Fuentemilla et al., 2010). This frontal theta increase is known as the frontal midline theta (FMT) and may, in part, be related to hippocampal activity (Klimesch, 1999; Mitchell et al., 2008). During recollection, theta activity has also been shown to mediate dynamic links between hippocampal and neocortical areas (Guderian et al., 2009). Alpha-band activity, on the other hand, has been associated with corticocortical and thalamocortical networks that are thought to reflect alertness, memory performance, and attention demands (Jensen et al., 2002; Hsieh et al., 2011; Klimesch, 2012). Decreases in alpha power (i.e., desynchronization) with increasing task difficulty reflect an inverse relation to the number of cortical resources allocated to task performance (Gevins et al., 1997; Babiloni et al., 2010). Measuring these neural oscillations pre- and post-training may aid in understanding how physical exercise impacts cognitive functions.

In rodents, voluntary wheel running has been associated with hippocampal neurogenesis and was positively correlated with synaptic plasticity (Vivar et al., 2012) and spatial pattern separation (Creer et al., 2010). In older adults, Erickson et al. (2009) found a triple association between aerobic fitness levels, hippocampal volume and memory functions, where higher aerobic fitness levels were associated with the preservation of left and right hippocampal volume and better performance on their spatial memory task (Erickson et al., 2009). Together, these studies supported the idea that exercise-induced hippocampal plasticity may improve learning and memory.

Recently, a cross-sectional study showed a relationship between aerobic fitness and neural rhythms in a Posner visuospatial attention task in high- and low-fitness young adults (Wang et al., 2015). Interestingly, high-fitness participants had faster reaction times (RTs) as well as greater beta and theta power during target processing. These findings indicated that aerobic fitness could be positively related to visuospatial attention capacity through the modulation of attentional processes. Some researchers have argued that even single bouts of physical exercise may be sufficient to improve memory performance (Roig et al., 2016) and attentional processes (Hogan et al., 2013). However, it remains unclear whether these exercise-induced improvements in WM and attention are linked to changes in neural oscillations.

Hence, we sought to examine how theta and alpha activity is modulated by a 4-month physical training regimen and aimed to determine the exercise-induced effects on brain dynamics in the frontoparietal network. To that end, we acquired EEG data from sedentary young adults during a delayed match-to-sample (DMS) WM task, which measured mnemonic discrimination of visual stimuli, akin to pattern separation, and a visual-attention search (VAS) task that did not require any recognition memory. We focused on theta (5–7 Hz) and alpha (9–12 Hz) oscillatory activity due to their association with WM and attention, respectively. Based on previous findings, we expected that our training intervention would increase aerobic fitness, enhance mnemonic discrimination, and as an indication of hippocampal activity change, increase theta power in the EEG. Particularly, for the exercise group, we expected faster RT and/or higher accuracy. Only the exercise group was expected to present changes in EEG spectral power reflected as a greater increase in theta power during the DMS task.

## Materials and Methods

### Subjects

Forty-three healthy, sedentary young adults (age range: 19–34 years, mean age: 25.33 ± 3.62 years, 23 females) were recruited for the study; one participant dropped out of the study, and two participants were excluded because of technical issues with the EEG recording. All subjects reported no signs of neurological or psychiatric illness and had a normal or corrected-to-normal vision. After providing informed consent, participants were pseudorandomly assigned either to an aerobic exercise group (*n* = 18) or a control group (*n* = 22), which were balanced in terms of age, sex, and fitness level (Table 1). Subjects received monetary compensation for their participation, and the experiment was carried out in accordance with the guidelines of the ethics committee of the Faculty of Medicine from the Otto von Guericke University Magdeburg (OVGU).

**TABLE 1:** Group demographics at baseline.

**Variables**	**Exercise group**	**Control group**	**One-way ANOVA**
Age	26.1 (4.0)	24.7 (3.3)	*F*(1,38) = 1.345, *p* = 0.253
Sex (f/m)	10/8	13/9	*X*^2^(1,39) = 0.051, *p* = 0.822
BMI	25.33 (5.71)	23.44 (3.29)	*F*(1,38) = 1.345, *p* = 0.198
VO_2_-RC	28.12 (4.93)	29.26 (4.02)	*F*(1,38) = 0.656, *p* = 0.423
(La^–^)_b_	4.77 (1.34)	5.12 (1.43)	*F*(1,38) = 0.647, *p* = 0.426

*Blood lactate [(La^–^)_*b*_ in g/mol]; oxygen uptake at respiratory compensation (VO_2_-RC in ml/min/kg); body mass index (BMI in kg/m^2^).*

### Intervention Protocol

#### Cardiovascular Training (Exercise Group)

The exercise group ran on a stationary treadmill ergometer three times per week for 16 weeks. Each participant received an individually optimized 45–75-min training set, including 5-min warm-up and 5-min cool-down periods. Under the supervision of sports scientists, participants monitored their heart rate (HR) during their workouts and exercised at intervals with an intensity range of 70–90% of maximum heart rate (HRmax). Individual training intensities were determined by target HRs, as estimated by the Karvonen method (Karvonen et al., 1957), and verified to HR levels at the individual anaerobic threshold, as indicated by lactate measures.

#### Walking (Control Group)

The control group walked on the treadmill twice a week, which maintained variables such as social interaction, scheduling, and motivation similar to the exercise group, while not affecting their aerobic fitness. The control group walked for 10–12 min, with breaks in between, and maintained a maximum HR of approximately 50–60% HRmax. The setting and HR monitoring procedures were kept constant between the groups. Due to the low HR training zone, the maximum incline of the treadmill was 3% at a maximum walking speed of 4.5 km/h.

### Fitness Assessment

We assessed the consumption of oxygen (CPET Quark, COSMED, Italy) at the respiratory compensation point (VO_2_-RC) by graded maximal exercise testing on a treadmill ergometer. The initial speed of 3 km/h was increased every 2 min to a maximum of 6.5 km/h. During this time, the slope of the treadmill also increased from 0 to 18%. This testing occurred until a respiratory exchange ratio (RER) of 1:1 was reached, indicating exhaustion near the limit of the cardiorespiratory system. Here, the oxygen exhaustion criterion was defined by the uptake at respiratory compensation (VO_2_-RC) to better control for volitional effects. To assess lactate levels (Biosen C-Line, EKF Diagnostic, Magdeburg, Germany), capillary blood samples were taken from the earlobe during the resting state, at 2-min intervals during the fitness test, and 2 min after the maximum intensity. This fitness assessment was repeated after 4 weeks and again after 16 weeks, at the end of the intervention.

### Task Procedure

**FIGURE 1:**
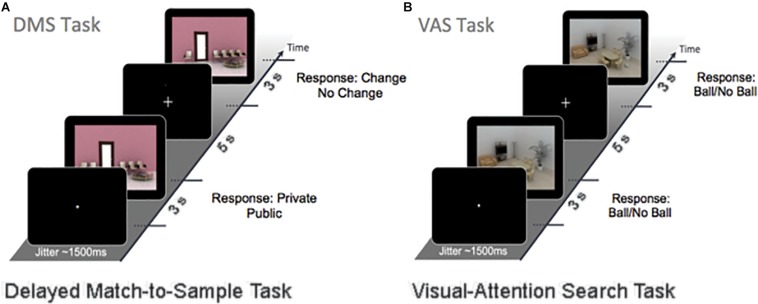
The working-memory experimental paradigm **(A)** is a DMS task (120 trials). A sample stimulus (encoding) and a probe stimulus (retrieval) were presented with a stimulus delay of 5 s (maintenance). Participants were instructed to respond to whether the sample stimulus was either private or public and to memorize the image. The probe stimulus was either the same (repeat item) or had small changes in the image (lure items) that needed to be detected. **(B)** The VAS task (60 trials). During this task, subjects were instructed to detect whether a target (i.e., ball) was present. The background image remained the same throughout the visual-attention paradigm.

Electroencephalography recordings were acquired a day before and 2–3 days after the end of the 4-month intervention, while participants performed 120 trials of a DMS WM task (Figure 1A) and 60 trials of a VAS task Figure 1B). The trials from both tasks were divided into 4 and 2 blocks, respectively, which were presented in a random sequence. During the DMS task, a sample stimulus and a probe stimulus were presented in succession for 3 s, separated by a 5-s delay (i.e., the maintenance phase). Subjects were instructed to answer as correct and quickly as possible whether the sample stimulus was a public or a private place and to spend the remaining time memorizing the image. The maintenance phase is critical for sustaining the WM item and is susceptible to interference from other external factors. Afterward, participants had to decide whether the probe image was identical to (i.e., repeat) or a modification (i.e., lure) of the sample image. All of the DMS stimuli were computer-generated indoor scenes, including 50% lure images. During the VAS task, participants were shown a similar indoor scene and were instructed to detect whether a target (i.e., spherical object) was present. The background image for the VAS remained the same throughout the different blocks, so neither encoding nor maintenance was required for this task.

### Data Acquisition and Processing

Continuous electroencephalogram (EEG) was acquired from 32 active electrodes mounted in an elastic cap (Brain Products GmbH, Germany) with a bandpass filter of 0.1–250 Hz and digitized at a rate of 500 Hz. We placed the electrodes according to the international 10–20 system. From the 32 electrodes, 2 electrodes were positioned at the external ocular canthi of each eye, and a vertical electrooculography (EOG) was placed below the left eye to measure horizontal and vertical eye movement, respectively. The left mastoid was used as the online reference, and all electrode impedances were kept below 5 kΩ. The skin under the electrodes was slightly abraded with a blunt needle, which was used to fill each electrode with electrolyte gel. MATLAB (MATLAB and Statistics Toolbox Release, 2012b, MathWorks, Inc., Natick, MA, United States) and its EEGLAB toolbox (Delorme and Makeig, 2004) were used for offline EEG data processing. The continuous EEG data were high-pass filtered at 0.1 Hz, low-pass filtered at 50 Hz, and referenced to the right mastoid. The data were segmented into 13-s epochs, including 1-s pre-stimulus as the baseline and 1-s post trial. Next, independent component analysis (ICA) was performed to correct for eye-related artifacts and excessive muscle activity. In addition, all trials were visually inspected, and those containing additional artifacts were excluded from further analysis. Participants included in the data analysis had less than 20% of their trials excluded after artifact correction.

### Time-Frequency Analysis

Event-related spectral perturbation (ERSP) was computed using algorithms from EEGLAB (Delorme and Makeig, 2004) and custom MATLAB scripts. Artifact-free data comprising 13-s segments were used for time-frequency analysis (TFA). Trial-by-trial event-related spectral power was calculated using Hamming window tapering with five cycles and 100 logarithmically spaced frequencies ranging from 3 to 20 Hz. For each frequency, event-related spectrum power at each time-frequency point was divided by the average spectral power in the pre-stimulus baseline period at the same frequency. These measures were normalized by taking the log value of the percentage of baseline activity (ERSP_%_) (Grandchamp and Delorme, 2011). By definition, the unit of ERSP_log_ is the decibel (dB), which is commonly used in the literature (Makeig, 1993; Cohen and Donner, 2013; Jiang et al., 2015).

**FIGURE 2:**
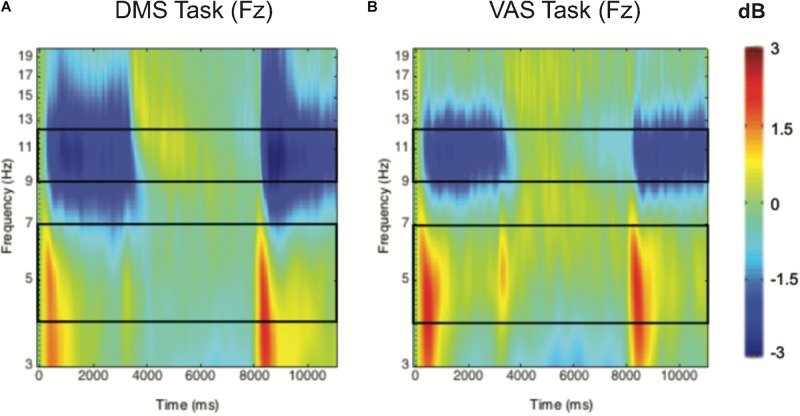
Group-averaged time-frequency 2D spectra (in dB) during a DMS task **(A)** and a VAS task **(B)** for the Fz electrode. Time (in ms) is indicated on the *x*-axis, with 0 ms defined as the onset of the sample stimuli and 8000 ms for the onset of the probe stimuli. Frequency (in Hz) is shown on the *y*-axis and is logarithmically scaled from 3 to 20 Hz. The event-related desynchronization (ERD) corresponds to negative values and is shown in blue, while the event-related synchronization (ERS) appears in red.

The mean ERSPs were calculated for two frequency bands of interest: theta (5–7 Hz) and alpha (9–12 Hz). These bands were chosen because of their involvement in visuospatial attention with the frontoparietal network (Wang et al., 2015). Because of frequency smearing after time-frequency decomposition, we used a width slightly different from the typically defined theta (4–8 Hz) and alpha (8–12 Hz) frequency bands (Klimesch, 1996). Narrower frequency bands were chosen to better characterize the changes in neural oscillations at those frequency bands (Hsieh et al., 2011). Additionally, electrodes were grouped into two different clusters with three electrodes each: frontal cluster (F3, Fz, and F4) and posterior cluster (P3, Pz, and P4). To measure if there is an exercise-induced effect, we focused on the sample probe. That is, power estimates from the frontal and posterior clusters were averaged across a section spanning the frequency band (see Figures 2A,B) and the time period of interest (0–3000 ms), and then subjected to statistical analysis.

### Statistical Analysis

All statistical analyses were performed using the statistical software SPSS (IBM Corps., IBM SPSS Statistics, V24, Armonk, NY, United States, 2016). First, we ran independent-samples *t*-tests to assess whether there were any differences between the groups at baseline (preintervention). To compare differences in categorical variables such as sex and age, we used the Chi-square test. There were no differences observed between the groups at baseline (Table 1).

Furthermore, to examine the possible effect of training on cognition, the behavior data were submitted to separate rmANOVAs (one ANOVA per condition), where the within factor was *time* (pre, post), and the between factor was *group* (exercise, control). As for the EEG data, these were submitted to two separate repeated measures ANOVAs (one rmANOVA per frequency band of interest). The factors in the rmANOVAs included *task* (DMS, VAS), *cluster* (frontal, posterior), and *time* (pre, post intervention) as within-subject factors and *group* (exercise, control) as a between-subjects factor. We did not include any variables as covariates since groups did not differ in demographics. When appropriate, the Greenhouse–Geisser correction was used to adjust the degrees of freedom when the sphericity assumption was violated. All alpha levels for significance were set at 0.05. When significant interactions were found with rmANOVA, *post hoc* independent *t*-tests were conducted.

### Correlations

To assess the possible relationship between exercise and cognition, we first calculated the intervention-related changes in performance, spectral power values, and fitness. In particular, accuracy, RT, and EEG spectral power differences were obtained by subtracting pre from post measures of EEG power. For a better estimation of aerobic fitness increase, a composite fitness score was calculated separately for pre- and postintervention fitness test data as a mean of the inverse *z*-scores of blood lactate level changes (%) and the *z*-score of VO_2_-RC changes (%). The bivariate (Pearson) correlations were calculated between changes in ERSP values, fitness scores, and behavioral performance to examine how fitness changes affect cognitive functioning after 4 months of exercise training.

## Results

### Participant Characteristics and Aerobic Fitness Assessment

Demographics and fitness levels for the exercise and control groups are reported in Table 1. As shown in Table 1, the groups were matched for age and sex and did not differ at baseline in body mass index (BMI), average blood lactate, and VO_2_-RC [*F*(1,38) < 1.35; *p* > 0.198].

To measure exercise-induced changes in fitness, we performed rmANOVAs with *time* (pre- and postintervention measures) as the within-subject factor and *group* (exercise and control) as the between-subjects factor. As seen in Table 2, rmANOVA revealed a time × group interaction for lactate [*F*(1,38) = 36.93; *p* < 0.001] as well as for VO_2_-RC [*F*(1,38) = 86.50; *p* < 0.001]. *Post hoc* paired *t*-tests showed an increase in fitness represented as a decrease in lactate [*t* (17) = −9.94; *p* < 0.001] and an increase in VO_2_-RC [*t* (17) = 2.27; *p* < 0.001] for the exercise group but not the control group [*t* (21) = −0.25; *p* = 0.809 and *t* (21) = −1.62; *p* = 0.121, respectively].

**TABLE 2:** Aerobic fitness measures pre and post intervention by group.

	**Exercise**		**Control**		
	**Pre**	**Post**	**Pre**	**Post**	
**Variables**	**M (SD)**	**M (SD)**	**M (SD)**	**M (SD)**	**rmANOVA**
(La^–^)_b_	4.77 (1.34)	3.05 (1.32)	5.13 (1.43)	5.50 (1.38)	*F*(1,38) = 36.926, *p* < 0.001
VO_2_-RC	28.12 (4.93)	32.60 (5.07)	29.26 (4.02)	29.32 (4.12)	*F*(1,38) = 86.496, *p* < 0.001

*Fitness measure: blood lactate [(La^–^)_*b*_ in g/mol]; oxygen uptake at respiratory compensation (VO_2_-RC in ml/min/kg).*

### Behavioral Data

**FIGURE 3:**
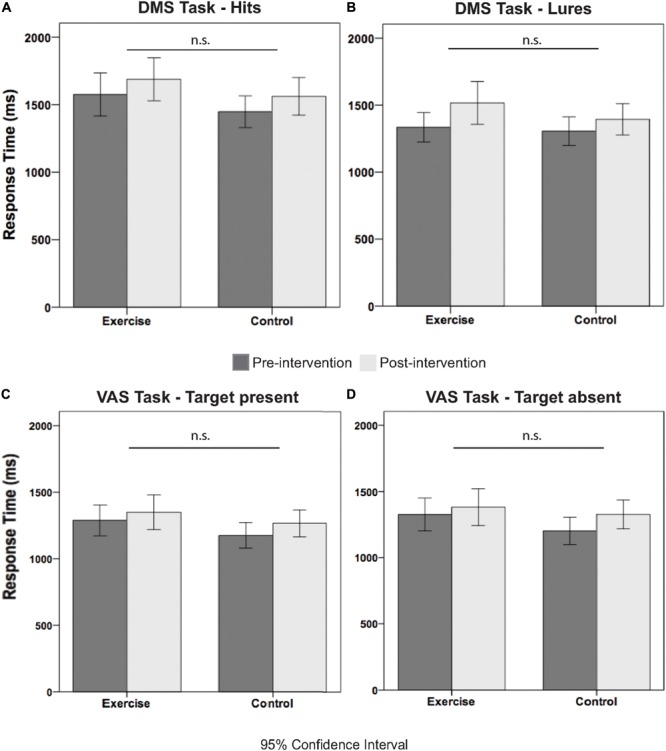
Illustrates the response time measures for pre and post intervention separated by group and condition. Specifically, **(A)** shows the RT pre and post intervention measures for hits, **(B)** for lures, **(C)** for target present, and **(D)** for target absent trials.

Table 3 illustrates the pre- and postintervention measures for accuracy and RT for each group. We considered performance values that were greater than 2.2 SDs from the mean as outliers, and these values were excluded from the statistical analysis. rmANOVAs revealed a main effect of time for all behavioral performance measures, for the exception of the correct response [*F*(1,36) = 2.00; *p* = 0.166; see Table 3]. Although the time × group interactions were all not significant (*F* < 1.96; *p* > 0.172), meaning that the difference in behavioral performance between the pre and post measures was not due to our intervention. Figures 3A–D further illustrates this point by showing the similar exercise-induced before and after performances between the exercise and the control group.

**TABLE 3:** Group mean (SD) for behavioral performance values pre and post intervention.

	**Exercise**		**Control**		**rmANOVA**	
	**Pre**	**Post**	**Pre**	**Post**	**Main effect**	**Interaction**
	**M (SD)**	**M (SD)**	**M (SD)**	**M (SD)**	**(Time)**	**(Time × group)**
**DMS**						
CHR	0.65 (0.12)	0.60 (0.12)	0.72 (0.11)	0.64 (0.17)	*F*_(1,32)_ = 8.956, *p* = 0.005	*F*(_1,32)_ = 0.305, *p* = 0.585
Hit RT	1576 (320)	1688 (321)	1448 (259)	1562 (306)	*F*_(1,37)_ = 10.099, *p* = 0.003	*F*_(1,37)_ = 0.000, *p* = 0.990
Lure RT	1335 (221)	1517 (321)	1306 (234)	1394 (258)	*F*_(1,37)_ = 16.224, *p* < 0.001	*F*_(1,37)_ = 1.938, *p* = 0.172
**VAS**						
CR	0.92 (0.04)	0.93 (0.02)	0.91 (0.03)	0.92 (0.03)	*F*_(1,36)_ = 1.996, *p* = 0.166	*F*_(1,35)_ = 0.022, *p* = 0.883
Target present RT	1288 (216)	1349 (247)	1142 (223)	1266 (214)	*F*_(1_,_35)_ = 7.945, *p* = 0.008	*F*_(1,35)_ = 0.311, *p* = 0.581
Target absent RT	1328 (233)	1397 (260)	1171 (234)	1328 (231)	*F*_(1,36)_ = 12.549, *p* = 0.001	*F*_(1,36)_ = 1.902, *p* = 0.177

*Included in Table 3 are the descriptive statistics and the results from a rmANOVA during the DMS and the VAS task. For the DMS task, accuracy was measured using hits minus false alarms, known as the corrected hit rate (CHR) and for the VAS task accuracy was measured by averaging the correct responses (CR) for both conditions (target present and absent).*

### Brain Dynamics and Behavioral Performance (Preintervention Measures)

**FIGURE 4:**
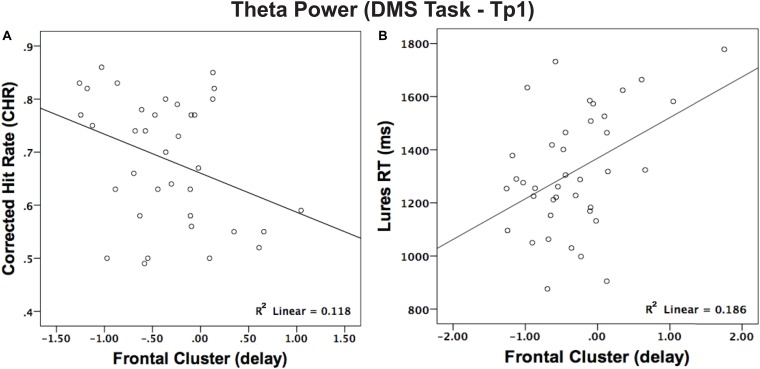
**(A)** Correlation between theta power in frontal regions and performance (corrected hit rate) during the maintenance phase (delay). Higher scores for corrected hit rates revealed a more prominent event-related desynchronization (ERD) for theta. **(B)** Correlation between theta power in frontal regions and RT (lure stimuli) during the maintenance phase. Shorter RTs for detecting lure items revealed greater ERD for theta during the maintenance phase (delay).

To test the fundamental relationship between spectral EEG power and behavioral performance, Pearson’s correlation analyses were performed on baseline measures across participants. For the DMS task, we did not find a correlation between FMT and behavioral performance during the encoding phase although there was a negative correlation between FMT and accuracy (corrected hit rate = hit−false alarm) during the maintenance phase [*r* (36) = −0.344; *p* = 0.040; Figure 4A], where better performance was associated with a greater decrease in theta power relative to baseline. Also, FMT correlated with RT, where faster detection of a lure was associated with greater decrease in theta power [*r* (39) = 0.431; *p* = 0.006; Figure 4B]. Notably, Figure 4B seems to have a high leverage point on the far right, although after obtaining the Mahalanobis distance, it was not excluded. Together, these correlations indicate that a greater decrease in theta during the maintenance conveys a more accurate and faster RT. As for the VAS task, correlations were performed between behavioral performance and posterior alpha power, which is where alpha power is most prominent and known to be related to attention (Frey et al., 2015). Our results showed a positive correlation between alpha power and RT during target absent trials [*r* (38) = 0.333; *p* = 0.041] and a marginal positive correlation for RT during target present trials [*r* (38) = 0.284; *p* = 0.084] but not for accuracy [*r* (40) = 0.020; *p* = 0.902].

### Exercise-Induced Spectral Power Changes (Pre- and Postintervention Measures)

**FIGURE 5:**
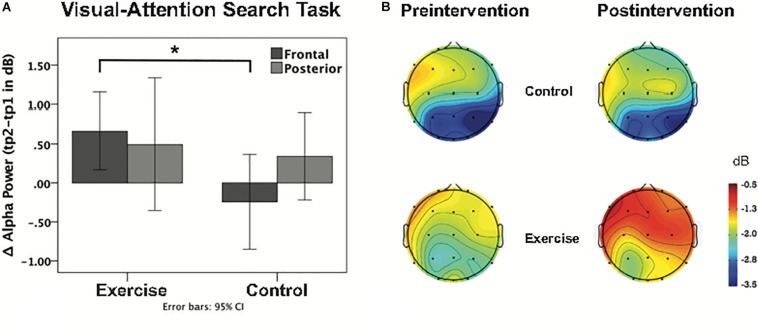
**(A)** Bar graph shows the intervention-induced alpha power change during the VAS task at frontal and posterior clusters for each group. Asterisk indicates *p* < 0.05 (uncorrected, 2-tailed *t*-test). **(B)** Topographical distribution of ERSP values for alpha-band activity, including the first 3 s of the sample stimuli presentation in both the pre and post intervention groups.

Repeated measures ANOVAs performed for the sample stimuli presentations (0–3 s) for theta power revealed a main effect of task [*F*(1,38) = 18.87; *p* < 0.001], no main effect of cluster [*F*(1,38) = 1.15; *p* = 0.290], and no main effect of time [*F*(1,38) = 0.01; *p* = 0.921]. The interaction between time and group was not significant [*F*(1,38) = 0.51; *p* = 0.480], as well as the task × cluster × time × group interaction [*F*(1,38) = 0.22; *p* = 0.645]. For the alpha band during the sample stimuli presentations (0–3 s), there was a main effect of *task* [*F*(1,38) = 25.09; *p* < 0.001], a main effect of *cluster* [*F*(1,38) = 17.76; *p* = 0.000], and no main effect of time [*F*(1,38) = 2.45; *p* = 0.126]. There was however, a significant *task* × *cluster* × *time* × *group* interaction [*F*(1,38) = 4.26; *p* = 0.046]. *Post hoc* independent *t*-tests showed no group difference for the posterior cluster [*t* (38) = 32; *p* = 0.751], while the frontal cluster showed a significant group difference [*t* (38) = 2.34; *p* = 0.025] during the VAS task, with increased alpha power after the intervention (see Table 4). This effect was specific to attention, as it appeared during the VAS task but not the DMS task [*t* (38) < 1.52; *p* > 0.136]. This EEG finding is further illustrated in Figures 5A,B in a histogram and a topographic map.

**TABLE 4:** Group mean (SD) values for theta and alpha power for frontal and posterior sites pre and post intervention.

	**Exercise**		**Control**		
	**Pre**	**Post**	**Pre**	**Post**	
	**M (SD)**	**M (SD)**	**M (SD)**	**M (SD)**	**Independent *t*-test**
**Theta**					
DMS (frontal)	−0.46 (0.83)	−0.34 (1.19)	−0.13 (0.76)	−0.08 (0.96)	*t* (38) = 0.068, *p* = 0.795
DMS (posterior)	−0.56 (1.44)	−0.33 (1.39)	−0.17 (1.46)	−0.39 (1.51)	*t* (38) = 2.315, *p* = 0.136
VAS (frontal)	0.20 (0.60)	0.14 (1.04)	0.33 (0.86)	0.28 (0.95)	*t* (38) = 0.000, *p* = 0.988
VAS (posterior)	−0.04 (1.38)	−0.03 (1.41)	0.21 (1.34)	0.01 (1.34)	*t* (38) = 0.253, *p* = 0.618
**Alpha**					
DMS (frontal)	−1.85 (1.63)	−1.89 (1.52)	−2.38 (1.79)	−2.39 (1.75)	*t* (38) = 0.005, *p* = 0.945
DMS (posterior)	−2.74 (2.73)	−2.46 (1.92)	−3.86 (2.29)	−3.62 (2.37)	*t* (38) = 0.013, *p* = 0.910
VAS (frontal)	−1.77 (1.62)	−1.10 (1.39)	−1.80 (2.16)	−2.04 (1.59)	*t* (38) = 5.465, *p* = 0.025
VAS (posterior)	−2.30 (2.66)	−1.81 (2.03)	−3.45 (2.33)	−3.11 (2.01)	*t* (38) = 0.102, *p* = 0.751

*Table 4 shows the frontal and posterior mean (SD) ERSP values for frontal and posterior theta- and alpha-band power. Both frequency-band power values are separated by group and further separated by task and time (pre and post intervention measures). Independent *t*-tests were conducted on differences in theta and alpha power (pre minus post intervention) between groups.*

**FIGURE 6:**
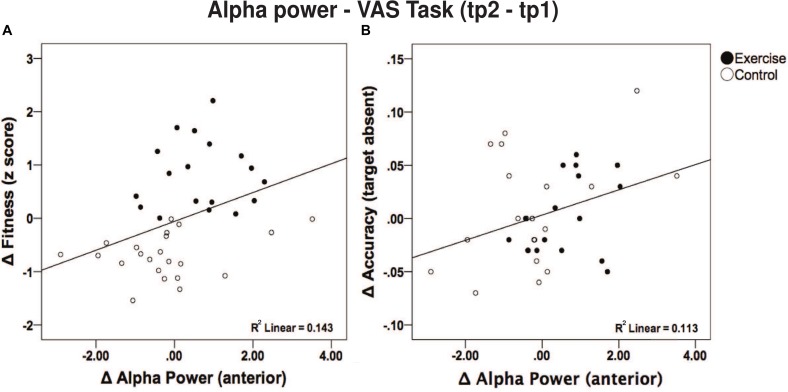
**(A)** Correlation between changes in alpha power in frontal regions and fitness score. A higher fitness score was related to a greater increase in alpha power. **(B)** Correlation between change in alpha power in frontal regions and correct response during the VAS (target absent trials) task.

Furthermore, given the significant *post hoc* results, we sought to examine a direct link between anterior alpha power changes with aerobic fitness changes and behavior performance changes (during the VAS task). Such correlations were performed irrespective of the group (i.e., across all subjects) since fitness changes were also present in the control group. Specifically, there was a positive correlation between changes in frontal alpha power and changes in aerobic fitness [*r* (40) = 0.379; *p* = 0.016; see Figure 6A]. Additionally, changes in frontal alpha power were also positively correlated with changes in accuracy [VAS task – see Figure 6B, target absent, *r* (37) = 0.336, *p* = 0.042; target present, *r* (39) = 0.302, *p* = 0.066]. Although a correlation between changes in aerobic fitness and changes in accuracy (*p* > 0.423) was absent. However, changes in aerobic fitness did correlate negatively with RT for the target present condition [*r* (38) = −0.360; *p* = 0.026] and marginally for the target absent condition [*r* (37) = −0.297; *p* = 0.074], yet failed to correlate with changes in alpha power (*p* > 0.345).

## Discussion

As anticipated, we observed an increase in aerobic fitness after a 4-month exercise intervention. The exercise intervention led to an increase in oxygen consumption at ventilatory anaerobic threshold, and a decrease in blood lactate threshold at maximum intensities. Contrary to our expectations, we did not observe intervention-related improvements in mnemonic discrimination or visual attention over time. Additionally, there were no effects of the exercise intervention on theta oscillations. However, the TFA revealed an increase in amplitude for frontal alpha power in the exercise group compared to that in the control group during the visual-attention-search (VAS) task. This overall change in alpha power positively correlated with aerobic fitness changes and changes in accuracy during the VAS task across all participants. Changes in fitness were further associated with RT behavioral performance in the VAS task as well.

### Preintervention Measures

A cross-sectional correlation analysis derived from baseline measures revealed that greater event-related desynchronization (ERD) in theta power was negatively correlated with memory performance (in the DMS task; Figure 4). Thus, stronger theta desynchronization in the maintenance phase was associated with better mnemonic discrimination and faster RT (in lure trials), which is in line with previous findings in the literature (Greenberg et al., 2015). Notably, the correlation between frontal theta power and performance was specific to lures (RT), potentially indicating that a decrease in frontal theta power associates with trials that require greater brain resources. In addition, the time-frequency representations (TFRs) for alpha power are in accordance with previous studies stating that alpha ERD occurs during a broad range of cognitive tasks, where harder tasks elicit a more negative ERD (Klimesch, 1999). Also, our findings revealed a positive correlation between alpha power and RT during target absent trials, where faster RT had greater ERD at posterior sites, further illustrating the importance of alpha desynchronization in attention processing. And, even though alpha power is most prominent in the parietal region, alpha ERD has also been observed in the frontal cortex during the performance of a selective attention task (i.e., Stroop task; Chang et al., 2015), indicating that alpha ERD reflects a top-down process.

### Exercise-Induced Changes

We observed an exercise-related increase in alpha power (i.e., weaker ERD), predominantly at the frontal electrodes during the VAS task (see Figure 5). Note that an increase in alpha power stems from a reduction in alpha ERD in the postintervention session. That is, overall subjects had similar performance levels, but the exercise group was characterized by reduced alpha desynchronization (i.e., less cortical activity) to accomplish the same task. Tentatively, we attribute this increase in alpha power to neural efficiency, specifically to an enhanced capacity for resource allocation involving visual attention. Our findings are compatible with the Ludyga et al. (2016) study in which they measured activity before and after a month of a cycling intervention, and also observed improvements in fitness performance and decreased cortical activity during exercise (Ludyga et al., 2016).

Furthermore, neuroimaging studies have demonstrated that subjects with higher WM and spatial skills have weaker frontoparietal activation during cognitive tasks (Rypma and D’Esposito, 1999). Similar results have been found in EEG studies where some have indicated that elite athletes (e.g., shooters, karatekas, and gymnasts) require “less” cortical activation (weaker ERD) in task-relevant brain areas than novices during sport-specific tasks (Ludyga et al., 2016) and a judgment-allocating task (Del Percio et al., 2009; Babiloni et al., 2010). This observation has been coined “neural efficiency” (Del Percio et al., 2010; Maffei et al., 2017). Studies that have taken aerobic fitness into consideration have come to similar conclusions (Hogan et al., 2015; Ludyga et al., 2016). For instance, a cross-sectional study performed by Hogan et al. (2013) found that preadolescents with lower fitness require more neural resources for a given task, which was reflected by an increased EEG coherence in comparison to the fit subjects.

Moreover, our results showed that participants with greater improvements in fitness had a larger increase in alpha power in the anterior regions (Figure 6A). Other cross-sectional studies have also found comparable correlations (Weinstein et al., 2012; Chu et al., 2016; Hyodo et al., 2016). An exercise intervention study in older adults found that higher aerobic fitness derived from the walking program was associated with greater changes in white matter integrity in the frontal and temporal lobes (Voss et al., 2013). Given these previous findings, it is congruent that we observed an increase in alpha power reflected in the anterior electrodes, which also correlated with changes in accuracy (Figure 6B). Notably, such correlations (alpha-fitness and alpha-performance) were existent across groups, implying that alpha power modulations may also be sensitive to other physiological changes. Fitness changes, for instance, might not only be driven by the intervention, but also other parameters such as daily routine activities, adaptability in training (Bonafiglia et al., 2016), and a genetic disposition to physical activity could also be playing a role (Bouchard, 2012). Nonetheless, only our exercise group showed less cortical activation to performed the VAS task compared to the control group, as shown by the increased alpha power after the intervention, which we attribute to neural efficiency.

Our study has some limitations. First, we had a limited sample size of 18 subjects comprising the exercise group. Further intervention studies will have to confirm our findings in larger samples, and it would be beneficial to lengthen the intervention duration to determine whether the training benefits transfer to hippocampal-dependent tasks and oscillations in young adults. On the other hand, our sedentary group was matched in age, sex, and baseline fitness level. Second, we did not have a tracker to measure outside activity; however, we encouraged all participants to avoid changes to their lifestyle outside the laboratory for the time of the intervention. The sedentary group of young adults who underwent our vigorous exercise training showed improvements from our 4-month training intervention, which we measured with not one but two fitness markers providing a more precise indication of the aerobic fitness change. Hence, the negative findings that we observed here were not due to an ineffective intervention.

Taken together, our study provides tentative evidence in support of cardiovascular exercise modulating oscillatory brain activity. Also, our findings support the possibility that aerobic training of sedentary young adults may influence neural dynamics underlying visual attention. However, we did not confirm our hypothesis that aerobic exercise would enhance theta oscillations and improve WM performance in a mnemonic discrimination task.

## Data Availability Statement

The raw data supporting the conclusions of this manuscript will be made available by the authors, without undue reservation, to any qualified researcher.

## Ethics Statement

The studies involving human participants were reviewed and approved by the Otto von Guericke University Magdeburg. The patients/participants provided their written informed consent to participate in this study.

## Author Contributions

AC performed the EEG experiments, analyzed EEG data, and wrote the manuscript. AB designed the study, performed the fitness research, analyzed fitness data, and edited the manuscript. ED designed the research and edited the manuscript.

## Conflict of Interest

The authors declare that the research was conducted in the absence of any commercial or financial relationships that could be construed as a potential conflict of interest.
